# Characterization of Heterogeneous MRSA and MSSA with Reduced Susceptibility to Chlorhexidine in Kuwaiti Hospitals

**DOI:** 10.3389/fmicb.2017.01359

**Published:** 2017-07-20

**Authors:** Leila Vali, Ali A. Dashti, Febine Mathew, Edet E. Udo

**Affiliations:** ^1^Department of Medical Laboratory Sciences, Faculty of Allied Health Sciences, Kuwait University Sulaibekhat, Kuwait; ^2^Department of Medical Microbiology, Faculty of Medicine, Kuwait University Sulaibekhat, Kuwait

**Keywords:** MRSA, MSSA, PFGE, MLST, spa typing, chlorhexidine

## Abstract

The objective of this investigation was to identify the lineages of MRSA and MSSA with reduced susceptibility to chlorhexidine in Kuwaiti hospitals. 121 clinical MRSA and 56 MSSA isolates were included in this study. Antimicrobial susceptibility testing was performed for a selection of agents including chlorhexidine and resistance genes were amplified and sequenced. PFGE, spa typing, and MLST were completed for a selection of isolates. The results showed SCC*mec* II, III, IV, and V were present in 0.8, 21.5, 69.4, and 8.3% of the MRSA isolates. *agr-1*_*Sa*_ was the most prevalent type in both MSSA (48%) and MRSA (54%). Forty-five percentage of MRSA contained *pvl* and 39% contained *lukE-lukD*, however, as many as 86% of MSSA contained *pvl* and 96.4% contained *lukE-lukD. qac* A-C genes were identified in 12.3% of MRSA, *nor*A was present in 82.6% and *bla*Z in 94.2%. Among MSSA only 5.4% harbored *qac*A, 83% contained *nor*A, and 91% *bla*Z. Multi-drug resistant ST239/t945 lineage containing a *qac* gene was the most identified *S. aureus*. However, other lineages, including ST772-MRSA-V/t4867/*pvl*(+)*qacC/smr* and non-*qac* harboring lineages of ST217-MRSAIV/t3244/*pvl*(–), ST34-MSSA/t161/*pvl*(+), ST5-MSSA/t688/*pvl*(+), ST5-MSSA/t4867/*nor*A(+), and ST672-MSSA/t003/*pvl*(–), also showed reduced susceptibility to chlorhexidine. The observed reduced susceptibility of non-*qac* dependent MSSA isolates to chlorhexidine suggests the involvement of other elements in promoting higher MBC (≥30 mg/L). Our results confirm that monitoring MSSA is essential as they may have the potential to survive low level biocide exposure.

## Introduction

*Staphylococcus aureus* (*S. aureus*) is a collection of multi-lineage Gram-positive cocci that is commensal to humans and is commonly found in the upper respiratory tract of 20~30% of general population. Yet *S. aureus* is a leading cause of bacteraemia (Stefani et al., 2012) in hospitals, and is the most common cause of necrotizing pneumonia, skin, and soft-tissue infections in community (Klevens et al., 2007; DeLeo and Chambers, 2009).

*S. aureus* contains mobile genetic elements (MGE), comprising of bacteriophages, pathogenicity islands, and transposons that carry genes encoding for antimicrobial resistance and virulence factors. Horizontal gene transfer of these mobile elements may result in strains that are increasingly pathogenic, epidemic, and highly resistant to antibiotics (Tong et al., 2015). It has been suggested that methicillin resistant *S. aureus* (MRSA) has evolved from methicillin-susceptible *S. aureus* (MSSA) via acquisition of Staphylococcal cassette chromosome *mec* (SCC*mec* types I–XI) (Baranovich et al., 2010; Petersdorf et al., 2015). Usually lineages that are important in hospital-acquired infections harbor SCC*mec* III element containing a large number of resistance genes (Ito et al., 1999). In contrast, SCC*mec* IV and V are frequently detected in isolates causing community acquired infections, although recently they have also been associated with hospital-acquired infections (Ito et al., 2001; Pereira et al., 2014).

According to the National Committee for Clinical Laboratory Standards (CLSI), the criteria for identifying MRSA is defined as *S. aureus* harboring *mec*A or *mec*C genes or phenotypically showing minimum inhibitory concentration (MIC) of oxacillin or cefoxitin ≥4 mg/L. The presence of *mec*A gene confers resistance to β-lactam antibiotics but not to penicillins.

MRSA and MSSA may contain *blaZ* gene expressing a β-lactamase enzyme that confers resistance to penicillins only. It is thought that the advantage of the presence of the β-lactam resistance mechanism mediated by *mecA* gene would keep the *bla* system active (Milheiriço et al., 2011). Other resistance genes that may be present in *S. aureus* include the multidrug efflux transporter *norA* that confers resistance to a broad spectrum of compounds, comprising of fluoroquinolones, quaternary ammonium compounds, efflux inhibitors such as reserpine, verapamil, and some dyes [ethidium bromide (EtBr), rhodamine, and acridines] (Roy et al., 2013). Also, proteins of both MFS (major facilitator superfamily) and SMR (the small multidrug resistant) family encode efflux-mediated resistance to a range of structurally unrelated cationic and lipophilic substrates across the cell membrane. In general, MFS encodes genes such as *qac*A, conferring resistance to a range of chemicals including ethidium bromide and chlorhexidine, while *qacB* confers resistance primarily to monovalent organic cations and some divalent compounds. Finally, *smr* genes consisting of *qacC* and *qacD* (Mayer et al., 2001) confer resistance to quaternary ammonium compounds (QACs) (Shamsudin et al., 2012) but not to chlorhexidine.

It is essential to use biocides and administer antimicrobial agents to reduce the bacterial load and decrease the probability of infections in hospitals. Chlorhexidine has been one of the most frequently used biocides in hospitals, at concentrations ranging from 0.5 to 4%, due to its broad spectrum of activity, tolerability, and safety record. It is commonly used as a skin antiseptic prior to clinical procedures, in dressings, hand disinfections and when bathing patients. The mechanism of action of chlorhexidine is known to be by binding to the negatively charged bacterial cell wall affecting the osmotic equilibrium of the cell.

While MIC and minimum bactericidal concentration (MBC) are commonly used to detect reduced susceptibility to chlorhexidine, there is, nevertheless, neither a defined standardized method nor consensus on the meaning of resistance to this agent (Horner et al., 2012). Recently, Morrissey et al. (2014) attempted to define breakpoints for chlorhexidine on the basis of normal distribution of MICs for a given bacterial species, known as the epidemiological cut-off value (ECOFF). ECOFF is described as the upper limit of the normal MIC distribution for chlorhexidine for a specific species and not the likelihood of treatment failure and clinical breakpoints as it is applicable for antibiotics. In general, the advised dose of chlorhexidine usage is several times higher than the MBC, yet, if chlorhexidine concentration reaches sub-lethal levels over time (Bloomfield, 2002), those isolates with reduced susceptibility to chlorhexidine will remain viable, survive, and possibly persist.

This study identifies the characteristics of lineages of MRSA and MSSA with reduced susceptibility to chlorhexidine in Kuwaiti hospitals and supports the notion that MSSA should be considered as an important agent of infection among hospitalized patients.

## Materials and methods

### Bacterial isolates

One hundred and twenty one MRSA and 56 MSSA were randomly obtained from Kuwaiti *S. aureus* Reference Laboratory in 2013. Tables 1, 2 show the origin of the isolates. Based on the data provided by the MRSA Reference Laboratory all isolates were non-duplicates from single patients. It is noteworthy to mention that our collection contained colonizing as well as infecting isolates. The isolates were confirmed in our laboratory as *S. aureus* by Gram stain, morphology, catalase, and coagulase tests. PBP2 was detected with a slide latex agglutination test (Oxoid, Besingstoke, UK).

**Table 1:** Clinical MRSA and MSSA isolates obtained from hospitals.

**Hospital**	**No. of MRSA**	**No. of MSSA**
Adan	10	0
Amiri	16	2
Razi	6	0
Sabah	30	11
Armed Forces	0	7
Chest	1	5
Farwaniya	24	3
Ibn Sina	0	1
Jahra	1	2
Maternity	13	4
Mubaarak	20	17
Unknown	0	4
Total	121	56

**Table 2:** Sources of isolates in this study.

**Specimen**	**No. of MRSA**	**No. of MSSA**
Abscess	7	0
Broncho Alveolar Lavage (BAL)	0	1
Blood	7	8
Ear	5	3
Eye	4	2
Fluid	5	1
Groin	11	3
High Vaginal Swab	1	3
Nasal	26	8
Catheter Tip	2	1
Pus	14	2
Rectal Swab	1	0
Skin	12	4
Sputum	1	2
Suction Tip	0	2
Swab	6	1
Thigh Abscess Pus	0	1
Tissue	2	2
Throat swab	1	1
Tracheal	2	1
Umbilical Swab	1	1
Urine	2	0
Wound	10	5
Unknown	1	4

### Susceptibility testing

Antibiotic susceptibility testing was performed by disc diffusion method, when applicable, following the Clinical Laboratory Standards Institute (2014) recommendations. The bacterial suspension (the final turbidity of a 0.5 McFarland Standard) was spread over the Mueller-Hinton agar uniformly and the antimicrobial discs were dispensed onto the agar plates using the disk dispenser and incubated overnight at 35°C. The antibiotic agents tested were penicillin (10 μg), cefoxitin (30 μg), ciprofloxacin (5 μg), clindamycin (2 μg) chloramphenicol (30 μg), erythromycin (15 μg), fusidic acid (10 μg), gentamicin (10 μg), kanamycin (30 μg), linezolid (10 μg), mupirocin (200 μg), rifampicin (5 μg), tetracycline (30 μg), teicoplanin (30 μg), tigecycline (15 μg), trimethoprim (5 μg). Inducible clindamycin resistance was detected with double-disk diffusion test (D-test). The diameter of zone of inhibition was measured (mm) and interpreted as recommended by EUCAST (2016) or CLSI guidelines (2014). MIC was determined by agar dilution method following CLSI recommendations for vancomycin (breakpoint >8 mg/L) and mupirocin (low-level resistance 8–64 mg/L and high-level resistance ≥512 mg/L). Bacterial growth above the breakpoint concentrations confirmed resistance to the relevant antibiotic. *S. aureus* ATCC 25923 was used as the control for disk diffusion and *S. aureus* ATCC 29213 for MIC assays. Isolates that showed resistance to at least three classes of antibiotic were considered as multi-drug resistant (MDR).

### Minimum inhibitory concentrations (MIC) and minimum bactericidal concentration (MBC) of chlorhexidine

Reduced susceptibility to chlorhexidine by MIC and MBC was based on the method described by Morrissey et al. (2014) and Furi et al. (2013). In this study the MIC for chlorhexidine digluconate (CH-; 100 mg/mL in water; C9394; Sigma-Aldrich, St. Louis, MO, USA) was determined using broth microdilution method with doubling concentration of chlorhexidine and starting inocula of 1 × 10^5^ CFU/mL. MBC was determined by subculturing 10 μL from each well without visible bacterial growth on Mueller-Hinton agar plates and incubating them at 37°C for 48 h. The first chlorhexidine dilution plate yielding three colonies or fewer was determined as MBC.

Interpretation of the results was based on the ECOFF, which is the upper limit of the normal MIC distribution for a given antimicrobial agent and a given species. For *S. aureus* and chlorhexidine MIC ≥ 4 mg/L and/or MBC ≥ 30 mg/L have been proposed.

### DNA isolation

Total genomic DNA for PCR and sequencing was extracted using the DNeasy Blood & Tissue Kit (Qiagen Valencia, CA, USA) according to the manufacturer's instructions.

### Detection of resistance and virulence genes

PCR was performed with HotStar *Taq* polymerase (Qiagen) according to the manufacturer's instructions and specific oligonucleotide primers for detection of the following genes: *mecA, mecC*, SCC*mec, agr* locus (*agr-1*_*Sa*_ to *agr-4*_*Sa*_), *lukE*-*lukD, lukS-PV*, and *lukF-PV, norA, qacA/B, qacC/qacD, qacG, qacH, blaZ, mupA, vanA, aac6*′*/aphD* (Oligonucleotide primers are listed in Supplement 1). Amplified PCR products were purified with Qiagen purification kit (Qiagen Valencia, CA, USA) according to the manufacturer's instructions and both strands were sequenced by automated AB13100 DNA sequencer (Applied Biosystems, Carlsbad, CA, USA) system. The BLAST program of the National Centre for Biotechnology Information (http://www.ncbi.nlm.nih.gov) was used to search and compare databases for similar nucleic acid sequences.

### *spa* typing

Based on the sequence analysis of polymorphic region X of protein A, a highly effective subtyping method for *S. aureus* is *spa* typing (Deurenburg et al., 2007). Amplification and sequencing of the variable region of the protein A gene, the sequence analysis and *spa*-type assignment were carried out as previously described (Harmsen et al., 2003). The X region of the *spa* gene was amplified by PCR with primers 1095F (5′-AGACGATCCTTCGGTGAGC-3′) and 1517R (5′-GCTTTTGCAATGTCATTTACTG-3′). DNA sequences were obtained with an ABI sequencer (Applied Biosystems). *spa* sequence types were determined with the database accessible via http://spa.ridom.de/ using BioNumerics version 7.1 (Applied Maths, Ghent, Belgium). The node distances between the spa types was calculated by minimum spanning tree method using Dice correlation (BioNumerics v.7.1.)

### Multi-locus sequence typing (MLST)

Genomic DNA was extracted from overnight cultures and MLST was carried out according to the protocol for *S. aureus* on the MLST website (http://www.mlst.net). The fragments of seven housekeeping genes (Supplement 1) *arcC, aroE, glpF, gmk, pta, tpi*, and *yqiL* were amplified and sequenced. Nucleotide sequences were aligned and trimmed using Bionumerics 7.1 software (Applied Maths, Ghent, Belgium). Allele numbers and sequence types (STs) were assigned by submitting sequences to the MLST website. MLST was performed on the isolates that contained *qac*A/B/C and on those isolates that showed reduced susceptibility to chlorhexidine.

### Pulse-field gel electrophoresis (PFGE)

Clinical isolates were typed by Pulse-Field gel electrophoresis (PFGE) with the CHEF-DR II electrophoresis cell after digestion with SmaI restriction endonuclease enzyme (Bannerman et al., 1995). The running parameters were as follows: initial pulse 5 s, final pulse 40 s, at 6 V/cm for 20 h at 14°C. The gels were stained with ethidium bromide and scanned. Profiles were analyzed by the unweighted pair method with arithmetic average (UPGMA) using BioNumerics v.7.1. The development of the algorithms necessary for the comparison of fingerprinting profiles of isolates was based on the Dice correlation coefficient. The hierarchic Cluster analysis and phylogenetic trees were subsequently analyzed with an optimization of 1.5% and a tolerance of 1.5%. Isolates were considered to belong to the same PFGE clone if their Dice similarity index was ≥85%.

### Qualitative real-time PCR amplification of *qac* genes

Real time qPCR was performed on *qac*A/B positive isolates. The total RNA was extracted using Qiagen RNeasy Kit (Qiagen Valencia, CA, USA) and cDNA was synthesized (High Capacity cDNA Reverse Transcription Kit, Applied Biosystems) and used as a template for qPCR amplification using SYBR Green method (EvaGreen qPCR Mix Plus, Solis Biodyne) using the primers described previously (Furi et al., 2013). Two TaqMan probes with two different fluorophores at the 5 = end and a minor groove binder (MGB) at the 3 = end (Applied Biosystems, United Kingdom) were used in order to distinguish between *qacA* and *qacB*. Qualitative real-time PCRs were performed in a Light Cycler 480 system (Roche Diagnostics, Germany). Melt curve analysis was performed along with the amplification protocol to determine if non-specific products were amplified during the reaction. The Ct (cycle threshold) value and melting curve analysis was used to confirm the presence of one peak and one product. Further agarose gel analysis (3%) was performed to confirm the amplification of a single PCR product.

### Statistical analysis

*P*-values were calculated using the *t*-test to determine whether differences between variants were significant using the unpaired two-tailed Mann-Whitney *U* (IBM SPSS version 23).

## Results

### Characterization of *S. aureus* isolates

**Figure 1:**
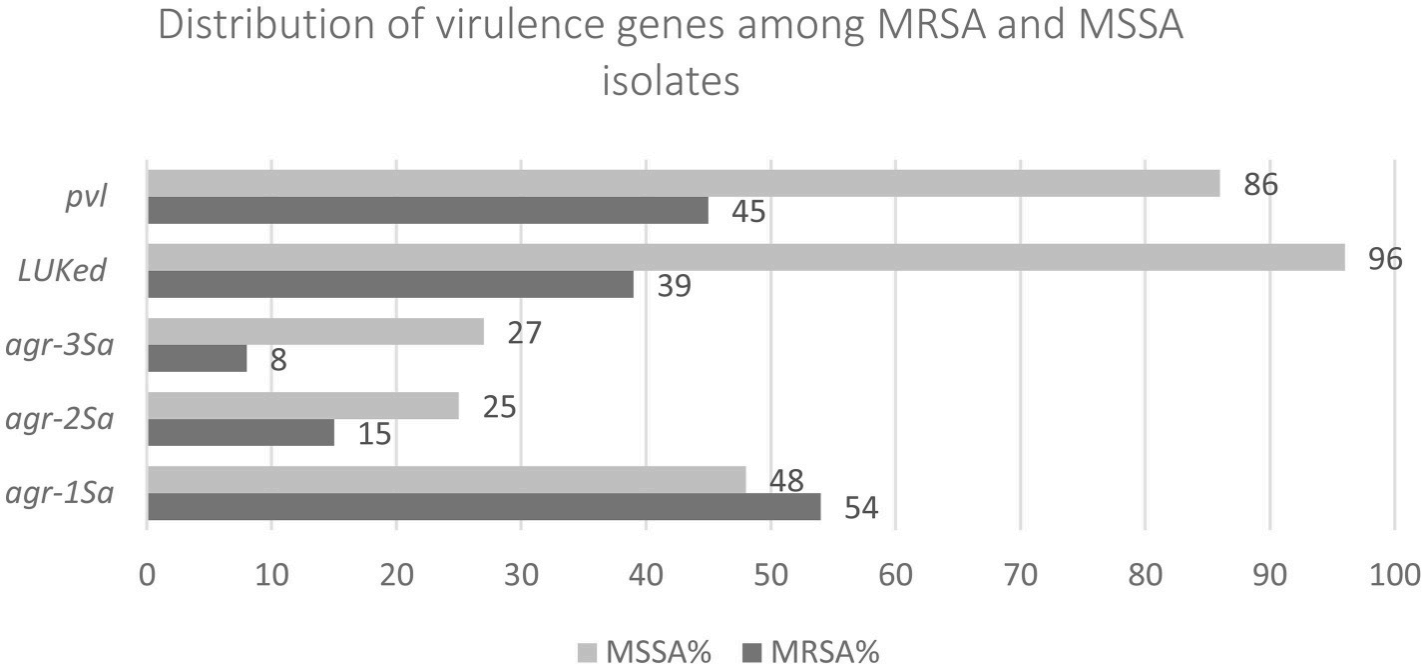
The percentage of virulence genes detected in MSSA isolates is higher than MRSA.

All 121 MRSA isolates were *mecA* positive and consisted of 1 (0.8%) SCC*mec* II, 26 (21.5%) SCC*mec* III, 84 (69.4%) SCC*mec* IV, and 10 (8.3%) SCC*mec* V. *mec*C was not detected. Sixty-five (54%), 18 (15%), and 10 (8%) contained *agr-1*_*Sa*_, *agr-2*_*Sa*_, and *agr-3*_*Sa*_ respectively, while 28 (23%) were *agr* negative. *pvl* was present in 45% (*n* = 55) while 39% (*n* = 47) contained *lukE*-*lukD* (Figure 1).

Among MSSA, *agr-1*_*Sa*_, *agr-2*_*Sa*_, and *agr-3*_*Sa*_ were present in 27 (48%), 14 (25%), and 15 (27%) of the isolates respectively. *agr-4*_*Sa*_ was not detected. 86% (*n* = 48) contained *pvl* and 96.4% (*n* = 54) contained *lukE*-*lukD* (Figure 1).

### Spa typing

Among the MRSA isolates, 36 known and two unknown *spa* types were identified: *spa* types t044 and t223 had the highest prevalence at 9.1% (*n* = 11) and 8.3% (*n* = 10) respectively. Among MSSA there were 37 known and 1 unknown *spa* types, t945 (9% *n* = 5 isolates) was the most prevalent. Altogether there was a two-fold variation in *spa* types among MSSA (38/56) vs. MRSA isolates (38/121) (68 vs. 31%) (*p* = 0.015). The common *spa* types shared among MRSA and MSSA were t002, t127, t223, t267, t304, t306, t688, t945, t3244, and t4867.

**Figure 2:**
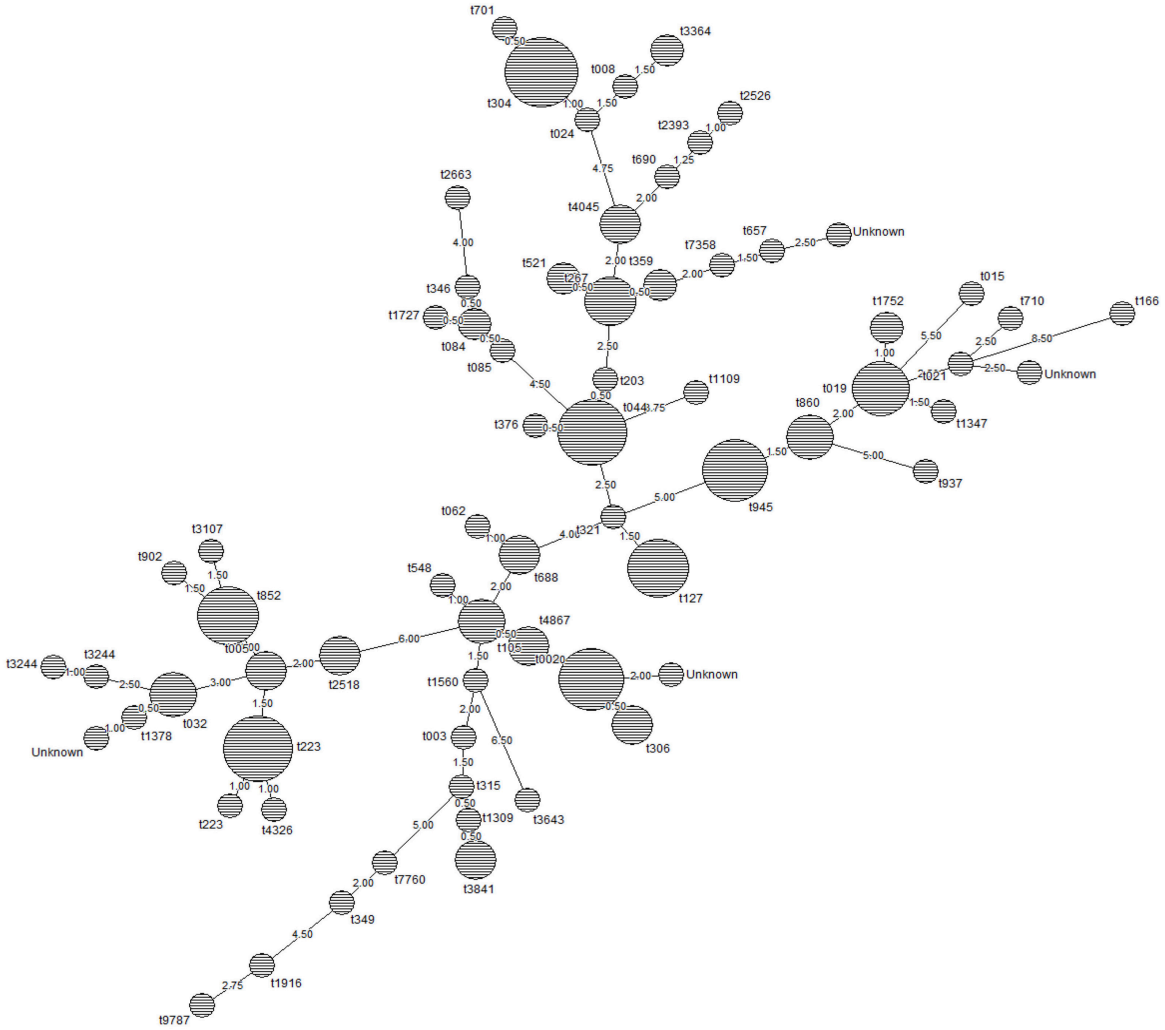
Relationship between *spa* types of MSSA and MRSA isolates in this study. Node size is proportional to spa type frequency and line length is proportional to the number of mutational steps between the types. The node distances between the spa types was calculated by minimum spanning tree method using Dice correlation (BioNumerics v.7.1.). Based on the default setting of 1%, distance intervals are created that are converted into distance units. For example two entries when having a similarity of 99.6% will have a distance of 0 (interval 100–99% = distance 0). Two entries that have a similarity of 98.7% will have a distance of 1 (interval 99–98% = distance 1).

Figure 2 shows the node distances between spa types based on clustering by minimum spanning tree method using Dice correlation (BioNumerics v.7.1.). Node size is proportional to spa type frequency and line length is proportional to the number of mutational steps between the types.

MRSA- *pvl* (+) isolates belonging to *spa* types t044 (*n* = 7), t019 (*n* = 5), t852 (*n* = 5) were the most prevalent, followed by t105 (*n* = 3); t002, t005, t945, t2518, and t3107 (*n* = 2 each); t024, t032, t086, t127, t548, t657, t690, t902, t1347, t1560, t1752, t3244, t4867, and t7358 (*n* = 1 each).

*pvl* (+) MSSA isolates were t945 (*n* = 4), t267 (*n* = 3), and t4045 (*n* = 3) followed by t127, t304, t306, t359, t688, t3841, and t4867 (*n* = 2 each); and t002, t015, t021, t062, t084, t085, t166, t315, t346, t349, t376, t521, t701, t710, t1109, t1727, t2393, t2526, t2663, t3244, t3643, t7760, t9787 (*n* = 1 each).

### Detection of biocide resistance genes

**Figure 3:**
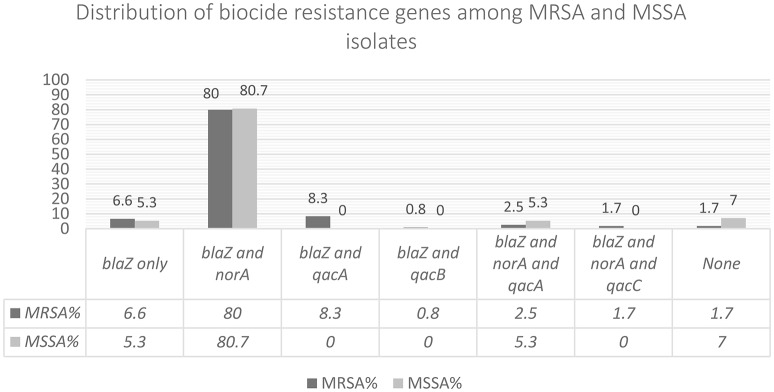
In this figure the combination of biocide resistance genes observed among the isolates has been shown.

Figure 3 shows the prevalence of combinations of biocide resistance gene observed in this study. *qac* genes were identified in 15 MRSA isolates (12.3%) of which 1 contained *qacC* (*smr*). Based on real time qPCR (Supplement 2), 13 isolates harbored *qacA* and 1 contained *qacB. norA* was present in 82.6% (*n* = 100), and *blaZ* in 94.2% (*n* = 114). Among MSSA, only 3 isolates (5.4%) harbored *qacA* gene. *norA* was present in 83% (*n* = 47), and *blaZ* in 91% (*n* = 51). We could not identify any statistically significant differences between the occurrences of either *blaZ* or *norA* in MRSA and MSSA isolates, however, MRSA was more likely to contain *qacA* gene than MSSA (*p* < 0.05).

### Antimicrobial resistance patterns

Table 3 shows the percentage of resistant isolates to the tested antimicrobial agents. For MRSA all isolates were resistant to oxacillin and cefoxitin and susceptible to rifampicin, tigecycline, teicoplanin, and linezolid. There were two vancomycin resistant MRSA isolates with MIC = 16 mg/L, typed as: (R15): ST239-MRSA-III/t860/*pvl*(–) *qacA* (+) and (S52): ST772-MRSA-V/t4867/*pvl*(+)*nor*A(+)*qacC*(+) (Table 4). PCR experiments using *van*A primers did not yield any bands. All MSSA isolates were susceptible to rifampicin, mupirocin, linezolid, tigecycline, teicoplanin, oxacillin, and cefoxitin. All isolates that were resistant to both gentamicin and kanamycin contained *aac6*′*/aphD* gene encoding the aminoglycoside-modifying enzymes. 44.6% of MSSA and 62.8% of MRSA were MDR.

**Table 3:** Percentage of resistance to each antimicrobial agent tested.

**Antimicrobial agent**	**No. resistant MRSA (%)**	**No. resistant MSSA (%)**
Penicillin	121 (100)	47 (84)
Cefoxitin	121 (100)	0
Ciprofloxacin	40 (33)	14 (25)
Chloramphenicol	2 (1.7)	0
Clindamycin (C)^a^	35 (29)	16(29)
Clindamycin (I)^b^	13 (11)	0
Erythromycin	49 (40)	16 (29)
Fusidic Acid	24 (20)	5 (9)
Gentamicin	41 (34)	38 (68)
Kanamycin	52 (43)	27 (48)
Mupirocin (HLR)^c^	4 (3.3)	0
Mupirocin (LLR)^d^	11 (9)	0
Tetracycline	34 (28)	16 (29)
Trimethoprim	34 (28)	7 (7)
Vancomycin	2 (1.7)	0

a (C) Constitutive resistance to clindamycin;

b (I) Inducible resistance to clindamycin;

c (HLR) High level resistance to mupirocin;

d *(LLR) Low level resistance to mupirocin*.

**Table 4:** Typing characteristics and antimicrobial profile of qac(+) MRSA and MSSA and qac(–) isolates with reduced susceptibility to chlorhexidine.

**Key**	**ST**	**Spa**	**SCC*mec***	**Specimen**	**Hospital**	***qac***	***nor*A**	***bla*Z**	***pvl***	***LUKED***	***agr***	**CHX MBC(mg/L)**	**Antibiotic resistance**
R-3	22	t4326	IV	Groin	MUBARAK (ICU)	–	+	+	–	–	1	30	Pen, Gen, Kan, Ery, Cli ^I^, Tri, Cip
R-4	22	t852	IV	Nasal	MUBARAK (ICU)	–	+	+	+	–	1	30	Pen, Gen, Kan, Ery, Cli ^C^, Tri, Cip
R-12	22	t852	IV	Eye	MATERNITY	–	+	+	+	–	1	30	Pen, Gen, Kan, Tri
R-13	22	t902	IV	Nasal	SABAH (PICU)	–	–	+	+	–	1	30	Pen, Gen, Kan, Tri
R-15	239	t860	III	Groin	MUBARAK (Surgical)	A	–	+	–	+	1	15	Pen, Gen, Kan, Ery, Cli^C^, Tet, Fus, Cip, Van
R-21	239	t945	III	Nasal	SABAH (ICU)	A	–	+	–	+	1	30	Pen, Gen, Kan, Ery, Cli^C^, Tet, Fus, Cip,Mup^LLR^
R-38	239	t945	III	Catheter Tip	SABAH (Internal)	A	–	+	–	–	1	7.5	Pen, Gen, Kan, Ery, Cli^C^, Tet, Fus, Cip,Mup^LLR^
R-45	239	t860	III	Groin	MUBARAK (Surgical)	A	–	+	–	+	1	30	Pen, Gen, Kan, Ery, Cli^C^, Tet, Fus, Cip, Mup
R-53	239	t860	III	Blood	MATERNITY (NICU)	A	+	+	–	+	1	30	Pen, Gen, Kan, Ery, Cli^C^, Tri, Fus, Cip,Mup^HLR^
R-62	5	t002	II	Nasal	AMIRI (Internal)	–	+	+	–	+	2	0.94	Pen, Gen, Kan, Ery, Cli^C^, Cip, Mup^HLR^
R-67	80	t044	IV	Abscess	AMIRI	–	+	+	+	+	1	1.875	Pen, Kan, Tet, Fus, Mup^HLR^
R-84	239	t945	III	Blood	SABAH (ICU)	A	+	+	+	+	1	60	Pen, Gen, Kan, Ery, Cli^C^, Tet, Fus, Cip, Mup^LLR^
R-87	80	t044	IV	Tissue	SABAH (PICU)	–	+	+	+	+	1	1.875	Pen, Gen, Kan, Ery, Cli ^I^, Tri, Cip, Mup^LLR^
R-101	239	t945	III	Skin	SABAH (Internal)	A	+	+	+	+	1	30	Pen, Gen, Kan, Ery, Cli^C^, Tet, Fus, Cip
R-102	239	t860	III	Nasal	MUBARAK (Surgical)	A	–	+	–	+	1	7.5	Pen, Gen, Kan, Ery, Cli^C^, Tet, Fus, Cip,Mup^LLR^
R-103	239	t860	III	Pus	MUBARAK (Liver dialysis)	A	–	+	–	+	1	0.94	Pen, Gen, Kan, Ery, Cli^C^, Tet, Fus, Cip,Mup^HLR^
R-104	239	t860	III	Groin	MUBARAK (Surgical)	A	–	+	–	+	1	0.94	Pen, Gen, Kan, Ery, Cli^C^, Tet, Fus, Cip,Mup^LLR^,
R-105	239	t945	III	Groin	SABAH (SICU)	A	–	+	–	–	1	0.94	Pen, Gen, Kan, Ery, Cli^C^, Tet, Fus, Cip,Mup^LLR^
R-106	239	t860	III	Nasal	RAZI (Isolation Unit)	A	–	+	–	+	1	0.94	Pen, Gen, Kan, Ery, Cli^C^, Tet, Fus, Cip,Mup ^LLR^
R-107	239	t945	III	Tracheal	SABAH (Surgical)	B	–	+	–	+	1	0.94	Pen, Gen, Kan, Ery, Cli^C^, Tet, Fus, Cip,Mup^LLR^
R-110	239	t945	III	Sputum	RAZI (Surgery)	–	+	+	–	+	1	0.94	Pen, Gen, Kan, Ery, Cli^C^, Tet, Fus, Cip, Mup^LLR^
R-111	239	t945	III	Fluid	SABAH (Outpatient)	–	+	+	–	+	1	0.94	Pen, Gen, Kan, Ery, Cli^C^, Tet, Fus, Cip, Mup^LLR^
R-113	22	t2518	IV	Pus	FARWANIYA (Surgical)	A	–	+	+	–	1	0.94	Pen, Gen, Kan, Cip
S-7	239	t945	MSSA	Skin	SABAH (Internal)	A	+	+	+	+	1	30	Pen, Gen, Kan, Tob, Cip, Tri
S-19	1	t127	MSSA	High Vaginal Swab	MATERNITY	–	+	+	+	+	3	30	Pen, Kan
S-22	88	t4045	MSSA	Blood	MUBARAK	–	+	+	+	+	3	60	Pen, Kan, Tob
S-23	97	t359	MSSA	Blood	MUBARAK	–	+	+	+	+	1	30	Pen, Kan, Tob
S-24	5	t4867	MSSA	Ear	SABAH	–	+	+	+	+	2	60	Pen, Gen, Kan, Tob, Tri
S-25	1	t127	MSSA	Nasal	SABAH (Internal)	–	+	+	+	+	3	60	Pen, Gen, Kan, Tob
S-27	22	t223	MSSA	Eye	MUBARAK (Pediatric)	–	+	+	–	+	1	30	Pen, Tob, Kan
S-32	5	t002	MSSA	Skin	FARWANIYA (Pediatric)	–	+	+	+	+	2	30	Cip, Gen, Kan,Tob
S-35	15	t084	MSSA	High Vaginal Swab	MUBARAK	–	+	+	+	+	2	30	Pen, Gen, Kan, Tob
S-41	932	t304	MSSA	Sputum	SABAH (ICU)	–	+	+	+	+	3	30	Pen, Gen, Kan, Tob
S-42	672	t003	MSSA	Blood	FARWANIYA	–	–	+	–	+	1	>60	Pen, Kan, Tob, Tri
S-43	965	t062	MSSA	Sputum	–	–	–	–	+	+	2	30	Pen, Gen, Kan, Tob
S-44	239	t945	MSSA	Groin	–	–	–	+	+	+	1	>60	Pen, Gen, Kan, Tob, Ery, Cip
S-46	5	t688	MSSA	Nasal	ICU	–	–	–	+	+	2	60	Pen, Gen, Kan, Tob,
S-47	217	t3244	IV	Blood	AMIRI(ICU)	–	+	+	–	–	1	60	Pen, Gen, Kan, Tob, Ery, Cip, Tri
S-48	239	t945	MSSA	Groin	SABAH (Surgical)	–	–	+	+	+	1	60	Pen, Gen, Kan, Tob, Ery, Cip
S-49	97	t359	MSSA	High Vaginal Swab	MATERNITY	–	–	–	+	+	1	30	Kan, Tob, Tri
S-50	96	t521	MSSA	Suction tip	ARMED FORCES	–	–	+	+	+	3	30	Pen, Gen, Kan, Tob, Cip, Tri,
S-51	5	t4867	MSSA	Tracheal	SABAH (ICU)	–	+	+	+	+	2	60	Pen, Gen, Kan, Tob, Tri
S-52	772	t4867	V	Nasal Swab	SABAH (Surgical)	C	+	+	+	+	2	30	Pen, Gen, Kan, Tob, Cip, Tri, Van
S-53	34	t166	MSSA	Umbilical Swab	MUBARAK(NICU)	–	+	+	+	+	3	30	Pen, Kan, Tob, Tri
S-56	239	t945	MSSA	Groin	SABAH (Surgical)	A	+	+	+	+	1	30	Pen, Gen, Kan, Tob, Ery, Cip
S-57	239	t945	MSSA	Swab	SABAH (Surgical)	A	+	+	+	+	1	7.5	Pen, Gen, Kan, Tob, Cip

*Cip, Ciprofloxacin; Chl, Chloramphenicol; CHX MBC, Chlorhexidine Minimum Bactericidal Concentration; Cli^C^, Constitutive resistance to clindamycin; Cli^I^, Inducible resistance to clindamycin; Ery, Erythromycin; Fus, Fusidic Acid; Gen, Gentamicin; ICU, Intensive Care Unit; Kan, Kanamycin; Mup^HLR^, High level resistance to mupirocin; Mup^LLR^, Low level resistance to mupirocin; NICU, Neonatal Intensive Care Unit; Pen, Penicillin; PICU, Pediatric Intensive Care Unit; SICU, Surgical Intensive Care Unit; Tet, Tetracycline; Tri, Trimethoprim; Van, Vancomycin*.

### Mupirocin resistance in MRSA

We did not find any MSSA isolates resistant to mupirocin. However, 4 MRSA isolates showed high-level and 11 showed low-level resistance (Tables 3, 4). The presence of *mup*A gene was detected by PCR generating a 1.65 kb intragenic fragment for both resistant phenotypes. The four high-level mupirocin-resistant isolates were typed as (R-45, R-103): ST239-MRSAIII/t860/*qacA*(+), (R-53): ST239-MRSAIII/t860/*qacA*(+)/*nor*A(+), and (R-62): ST5-MRSAII/t002/*nor*A(+).

11 MRSA low-level mupirocin-resistant isolates were typed as (R-21, R-38, R-105): ST239-MRSAIII/t945/*qac*A(+), (R-84): ST239-MRSAIII/t945/*norA*(+)/*qac*A(+), (R-87): ST80-MRSAIV/t044/*nor*A(+), (R-102, R-104, R-106): ST239-MRSAIII/t860/*qac*A(+), (R-107): ST239-MRSAIII/t945/*qac*B(+), (R-110, R-111) ST239-MRSAIII/t945/*norA*(+). These isolates were collected from a variety of specimens from different hospitals (Table 4).

### *qac* genes, MLST, and chlorhexidine

For MRSA, MIC range was 0.5–2 mg/L with MIC_50_ = 1 mg/L and MIC_90_ = 2 mg/L, MBC range was 1–30 mg/L with MBC_50_ = 4 mg/L and MBC_90_ = 30 mg/L. For MSSA, MIC range was 0.5–60 mg/L with MIC_50_ = 1 mg/L and MIC_90_ = 2 mg/L. MBC range was from 4 to >60 mg/L with MBC_50_ = 30 mg/L and MBC_90_ = 60 mg/L. These results suggest that although more MRSA than MSSA isolates harbored *qac* genes (*n* = 15 vs. *n* = 3), MBC_90_ was higher for MSSA implying that the presence of *qac*A might not be significant in reducing susceptibility or prompting higher MBC to chlorhexidine.

**Figure 4:**
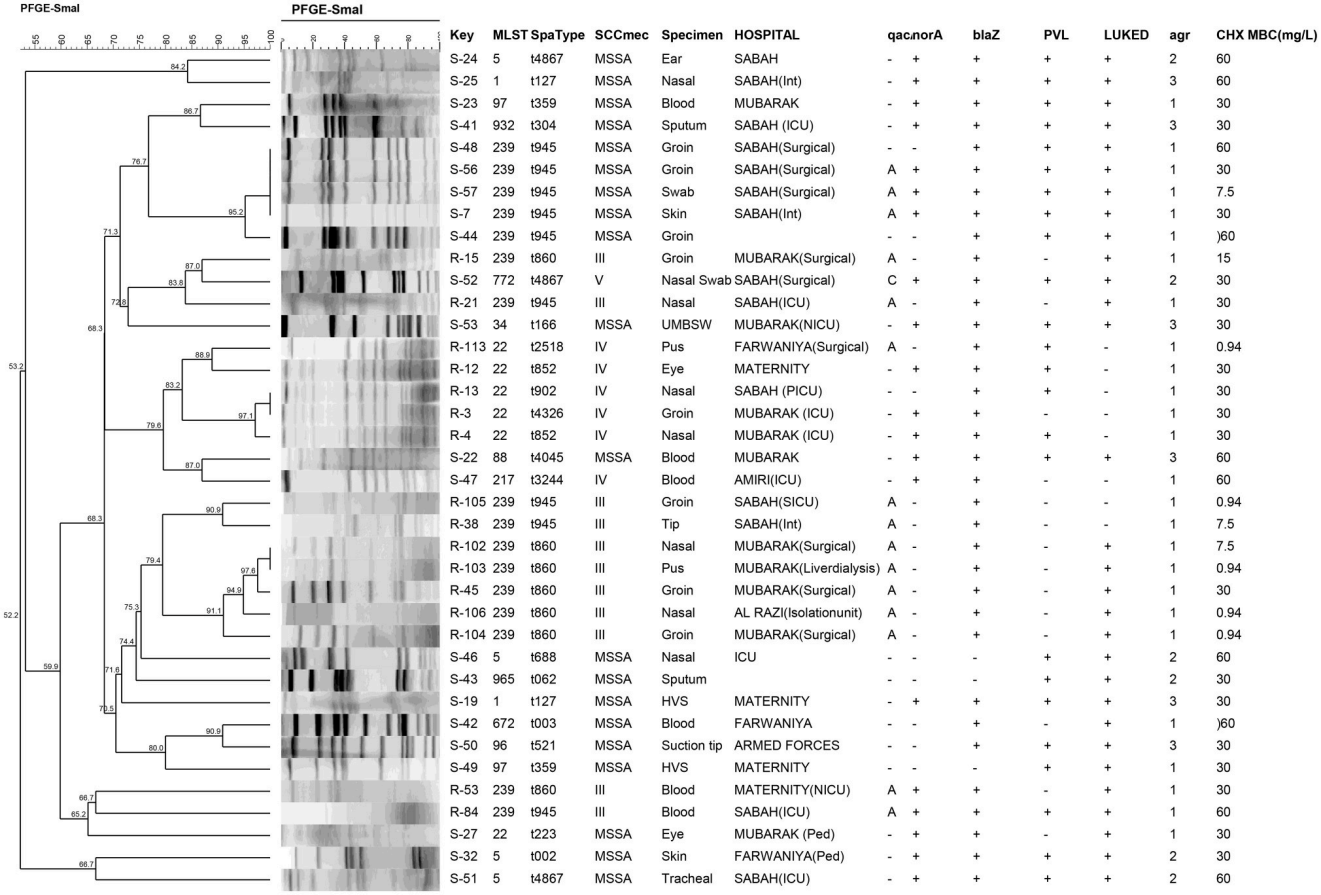
PFGE profiles of *qac*(+) MRSA and MSSA and *qac*(–) isolates with reduced susceptibility to chlorhexidine. The hierarchic Cluster analysis and phylogenetic tree after digestion with *Sma*I is shown. PFGE banding patterns were analyzed using DICE/UPGMA with an optimization of 1.5% and a tolerance of 1.5% generated by BioNumerics software (v.7.1). The percentage of similarities between the clusters are shown.

MLST and PFGE for *qac*(+) and those MRSA and MSSA isolates with MBC ≥ 30 mg/L are shown in Figure 4 and Table 4. Based on the suggested ECOFF, the reduced susceptibility to chlorhexidine did not exclusively depend on the presence of *qac* genes, however, MBC of chlorhexidine was higher for those *qac*A(+) isolates obtained from blood. For chlorhexidine, the median of MBC was statistically greater than the median of MIC (*p*-value < 0.0001). The median of MIC and MBC for *qac*A(+) isolates were higher than *qac*A(–) isolates (*p*-value 0.36 and 0.6, respectively). Results were analyzed using the unpaired two-tailed Mann-Whitney *U* (IBM SPSS version 23).

Moreover, there were *qac*A(+) isolates that did not display high MBC (MBC ≤ 7.5 mg/L) (Table 4) as well as *qac*A(–) MSSA isolates with reduced susceptibility to chlorhexidine (including S-53: ST34-MSSA/t161 isolated from an infected umbilical cord of a neonate, S-48: ST239-MSSA/t945, S-46: ST5-MSSA/t688, S-51: ST5-MSSA/t4867, S-42:ST672-MSSA/t003, S-19:ST1-MSSA/t127, S-35:ST15-MSSA/t084, S-22:ST88/t4045, S-50: ST-96/t521, S-49: ST97/t359, S-41: ST932-MSSA/t304 and S-43: ST965-MSSA/t062). Likewise *qac*A(–) MRSA isolates including ST22-MRSA-IV (with variable *spa* types), ST239-MRSA-III and ST217-MRSA-IV showed reduced susceptibility to chlorhexidine (Table 4, Figure 4).

The only *qac*B(+) isolate (R-107) in our collection was susceptible (MBC ≤ 1 mg/L) whereas the only *qac*C(+) isolate (S-52) showed reduced susceptibility to chlorhexidine (MBC = 30 mg/L).

### PFGE analysis

PFGE results for isolates with reduced susceptibility to chlorhexidine and those containing *qac* genes are shown in Figure 4. Cluster analysis was performed and phylogenic tree was prepared using dice similarities and unweighted matched-pair group which demonstrated diversity among the isolates. It also showed that none of the MSSA and MRSA isolates shared identical PF patterns.

## Discussion

This is the first study to identify heterogeneous genotypes of *qac* and non-*qac* harboring MRSA and MSSA with reduced susceptibility to chlorhexidine in Kuwait. The MBC_90_ of chlorhexidine for MSSA isolates was higher than that of MRSA, even though the number of isolates containing *qac* genes was higher in the MRSA group (*n* = 15 vs. *n* = 3). These results suggest that elements other than *qac* genes may be important for reducing susceptibility to chlorhexidine. One factor to consider would be the expression of the *agr* quorum-sensing system which is already known to influence virulence factor production but which may also affect antimicrobial resistance and metabolism through its interaction with other staphylococcal gene regulators (Tseng et al., 2015). In our study virulence factors were generally more prevalent in MSSA. Forty-five percentage MRSA and 86% of MSSA contained *pvl* while 39% of MRSA and 96.4% of MSSA contained *lukE*-*lukD* genes. Similar to a previous report (Warren et al., 2016) we also observed *qac*A(+) MRSA isolates were more likely to exhibit mupirocin resistance.

In this study the predominant resistance gene was *blaZ* which was present in 94.2% of MRSA and 91% of MSSA usually in combination with other biocide genes. Thirteen MRSA and only 3 MSSA isolates (5.4%) harbored *qac*A and 16 out of 18 (89%) *qac*(+) isolates were typed as ST239. ST239-MRSA-SCC*mec*III, also known as the hospital-acquired Brazilian/Hungarian clone, is one of the most successful lineages in the world owing its high rate of prevalence to an enhanced ability to form biofilm and a tendency to acquire genes that confer resistance to different classes of antimicrobials (Amaral et al., 2005). The most common reported spa type associated with ST239-MRSA is the ancestral spa type t037 (the plesiomorphic state) (Harris et al., 2010), but other spa types have also been linked to ST239. In this study spa types t945 and t860 were common between ST239-MRSA and ST239-MSSA however their PFGE banding patterns were dissimilar. In addition to ST239, other common lineages between MSSA and MRSA were ST5 and ST22. Similar observation but with different STs have been noted before (Crisóstomo et al., 2001). To determine whether these strains lost or gained Scc*mec* over time will require more genetic analysis.

Other MRSA lineages with reduced susceptibility to chlorhexidine were ST217, a single-locus variant of EMRSA-15, which is prevalent in Europe and India (Qi et al., 2005; Vignaroli et al., 2014; Bouchiat et al., 2015) and has been also detected from food-stuffs of animal origin (Lozano et al., 2009). Also the highly transmissible ST772-MRSA-V/t4867 (the Bengal clone) (Afroz et al., 2008) which has been detected in different countries (Fuentes et al., 2005; Nadig et al., 2012; Shore et al., 2014) including Kuwait (Boswihi et al., 2016). However, this is the first time *qac*C and reduced susceptibility to chlorhexidine has been identified in this infectious clone. *qac*C is a mobile gene that can be transferred between plasmids without the aid of insertion sequences or transposases also known as “DSO-gene-SSO” element (Wassenaar et al., 2016).

We also observed high MBC for chlorhexidine among MSSA lineages including ST34 and ST5; commonly linked to mother-to-infant transmission (Achermann et al., 2015; Benito et al., 2015), ST88-MSSA, a prevalent colonizer in Portugal and China (Qiao et al., 2014), ST97 (associated with food producing animals) (Lozano et al., 2011) and among other less reported lineages: ST96, ST932, and ST965.

Nevertheless in Staphylococci, *qac* genes may be associated with ecologically successful genetic lineages to retain a high level of endemicity and or epidemicity. In environments where biocide delivery is compromised, whether in the community or in hospitals, the possession of biocide resistance genes (*qac*) may contribute to clonal expansion (Cooper et al., 2012). Yet, high rate of the putative virulent *pvl* gene in MSSA clones with reduced susceptibility to chlorhexidine should be monitored with vigilance as they may lead to poor prognosis in patients.

Our overall conclusion from this study is that clinical MSSA isolates with reduced susceptibility to chlorhexidine are genetically diverse. Since MSSA infections are important globally and are not just a local problem, the typing information can be used to evaluate the significance of distribution of these lineages and their clonal relationships in other countries.

## Author contributions

LV, AD, and EU conceived and designed the study. LV and FM acquired the data. LV, FM, and EU analyzed the data. LV, AD, FM, and EU drafted and critically evaluated the manuscript. All authors approved the final version of the manuscript.

### Conflict of interest statement

The authors declare that the research was conducted in the absence of any commercial or financial relationships that could be construed as a potential conflict of interest.
