# Supplementary material for: Characterization of Heterogeneous MRSA and MSSA with Reduced Susceptibility to Chlorhexidine in Kuwaiti Hospitals
Source: Front Microbiol. 2017 Jul 20;8:1359. doi: 10.3389/fmicb.2017.01359 (PMC5517409; doi:10.3389/fmicb.2017.01359)
Supplement: Supplementary file 1 [file DataSheet1.DOCX]

**Supplement 1: Primers used in this study.**

**TABLE 1**: multiplex PCR for SCC*mec* typing

| Locus | Primer | Oligonucleotide sequence | Location | Amplicon Size (bp) | Specificity^e^ (SCC*mec* type) |
| --- | --- | --- | --- | --- | --- |
| A | CIF2 F2  CIF2 R2 | TTCGAGTTGCTGATGAAGAAGG  ATTTACCACAAGGACTACCAGC | 18398–18419^a^  18892–18871^a^ | 495 | I |
| B | KDP F1  KDP R1 | AATCATCTGCCATTGGTGATGC  CGAATGAAGTGAAAGAAAGTGG | 10445–10467^b^  10728–10707^b^ | 284 | II |
| C | MECI P2  MECI P3 | ATCAAGACTTGCATTCAGGC  GCGGTTTCAATTCACTTGTC | 42428–42447^b^  42636–42617^b^ | 209 | II,III |
| D | DCS F2  DCS R1 | CATCCTATGATAGCTTGGTC  CTAAATCATAGCCATGACCG | 38011–37992^a^  37670–37689^a^ | 342 | I,II,IV |
| E | RIF4 F3  RIF4 R9 | GTGATTGTTCGAGATATGTGG  CGCTTTATCTGTATCTATCGC | 45587–45607^c^  45829–45809^c^ | 243 | III |
| F | RIF5 F10  RIF5 R13 | TTCTTAAGTACACGCTGAATCG  GTCACAGTAATTCCATCAATGC | 59573–59594^c^  59986–59965^c^ | 414 | III |
| G | IS431 P4  pub110R1 | CAGGTCTCTTCAGATCTACG  GAGCCATAAACACCAATAGCC | 49963–49982^b^  50343–50323^b^ | 381 |  |
| H | IS431 P4  pT181 R1 | CAGGTCTCTTCAGATCTACG  GAAGAATGGGGAAAGCTTCAC | 29654–29673^c^  29976–29956^c^ | 303 |  |
| *mec*A | MECA P4  MECA P7 | TCCAGATTACAACTTCACCAGG  CCACTTCATATCTTGTAACG | 1190–1211^d^  1351–1332^d^ | 162 | Internal control |

^a^ Relative to accession no. AB033763, SCCmec type I (13).

^b^ Relative to accession no. D86934, SCCmec type II (12).

^c^ Relative to accession no. AB037671, SCCmec type III (12).

^d^ Relative to accession no. Y00688, mecA gene (34).

^e^ Loci G and H were included to distinguish variants IA from I and IIIA from III, respectively.

Oliveira D.C. and de Lencastre H. (2002) Multiplex PCR strategy for rapid identification of structural types and variants of the *mec* element in Methicillin-Resistant *Staphylococcus aureus*. *Antimicrob Agents Chemother*. 46: 2155–2161.

**TABLE 2:** Primers used in the multiplex PCR for SCC*mec* typing Multiplex Real-Time PCR for Rapid Staphylococcal Cassette Chromosome mec Typing ▿

[Chen](https://www.ncbi.nlm.nih.gov/pubmed/?term=Chen%20L%5BAuthor%5D&cauthor=true&cauthor_uid=19726600), L., [Mediavilla](https://www.ncbi.nlm.nih.gov/pubmed/?term=Mediavilla%20JR%5BAuthor%5D&cauthor=true&cauthor_uid=19726600) J. R., Oliveira D. C. , [Willey](https://www.ncbi.nlm.nih.gov/pubmed/?term=Willey%20BM%5BAuthor%5D&cauthor=true&cauthor_uid=19726600) B.M, de Lencastre H., and Kreiswirth B.N. (2009) Multiplex Real-Time PCR for Rapid Staphylococcal Cassette Chromosome mec Typing. [*J Clin Microbiol*.](https://www.ncbi.nlm.nih.gov/pubmed/19726600) 47:3692-706.

| Primer | Target | Sequence (5’-3’) | Amplicon Size (bp) |
| --- | --- | --- | --- |
| mecA-F  mecA-R | mecA | GCAATACAATCGCACATACATT  CCTGTTTGAGGGTGGATAGC | 148 |
| ccrB2-F  ccrB2-R | Type 2 ccr | CTCATGTTACARATACTTGCG  CCTTGATAATAGCCTTCTTGG | 107 |
| mecI-F  mecI-R | Class A mec | CGTTATAAGTGTACGAATGGTTTTTG  TCATCTGCAGAATGGGAAGTT | 126 |
| IS1272J-F  IS1272J-R | Class B mec | GAAGCTTTGGGCGATAAAGA  GCACTGTCTCGTTTAGACCAATC | 98 |
| ccrC-F  ccrC-R | Type 5 ccr | TCCAGTCTATAAAGGSTATGTCAG  ACTTATAATGGCTTCATGCTTACC |  |
| ccrB1-F  ccrB1-R  ccrB3-F  ccrB3-R | Type 1 and  Type 3 ccr | ACCACAAACACACTTAAAGATG  CAATTTCAAGTATTTGGTCCATAAC  AACACAACGAACACATTGAAAG  CGTATTTCTCAATCACATCAGC | 150  130 |
| ccrB4-F  ccrB4-R | Type 4 ccr | CGAAGTATAGACACTGGAGCGATA  GCGACTCTCTTGGCGTTTA | 134 |

| Gene | Primer | Oligonucleotide sequence (5’-3’)^a^ | Amplicon Size (bp)^a^ |
| --- | --- | --- | --- |
| agr_Sa_ | agr1-4_Sa_-1 | ATGCACATGG TGCACATGC |  |
| agr-1_Sa_ | agr1_Sa_-2 | GTCACAAGTACTATAAGCTG CGAT | 439 |
| agr-2_Sa_ | agr2_Sa_-2 | TATTACTAATTGAAAAGTGCCATAGC | 572 |
| agr-3_Sa_ | agr3_Sa_-2 | GTAATGTAATAGCTTGTATAATAATACCCA G | 321 |
| agr-4_Sa_ | agr4_Sa_-2 | CGATAATGCCGTAATACCCG | 657 |

**TABLE 3**: Primers used for *agr* typing

Lina G., Boutite F., Tristan A., Bes M., Etienne J., Vandenesch F. (2003). Bacterial competition for human nasal cavity colonization: role of Staphylococcal *agr* alleles. *Appl Environ Microbiol*. 69 18–23.

^a^ Nucleotide sequences and expected sizes of PCR products were derived from the published sequences of alleles agr-1_Sa_ to agr-4_Sa_ (GenBank accession numbers X52543, AF001782, AF001783, AF288215).

**TABLE 4**: Primers used for *spa* typing

Harmsen D., Claus H., Witte W., Rothgänger J., Claus H., Turnwald D., Vogel U.(2003). Typing of methicillin-resistant *Staphylococcus aureus* in a university hospital setting by using novel software for spa repeat determination and database management. *J Clin Microbiol*. 41 5442–8.

| Gene | Primer | Sequence (5’-3’) |
| --- | --- | --- |
| Protein A | 1095F  1517R | AGACGATCCTTCGGTGAGC  GCTTTTGCAATGTCATTTACTG |

**TABLE 5**: Primers used for MLST typing (https:// saureus.mlst.net/)

| Gene | Primer | Sequence (5’-3’) | Amplicon Size (bp) |
| --- | --- | --- | --- |
| Carbamate kinase *arcC* | arc up  arc dn | TTG ATT CAC CAG CGC GTA TTG TC  AGG TAT CTG CTT CAA TCA GCG | 456 |
| Shikimate dehydrogenase *aroE* | aro up  aro dn | ATC GGA AAT CCT ATT TCA CAT TC  GGT GTT GTA TTA ATA ACG ATA TC | 456 |
| Glycerol kinase *glpF* | glp up  glp dn | CTA GGA ACT GCA ATC TTA ATC C  TGG TAA AAT CGC ATG TCC AAT TC | 465 |
| Guanylate kinase *gmk* | gmk up  gmk dn | ATC GTT TTA TCG GGA CCA TC  TCA TTA ACT ACA ACG TAA TCG TA | 417 |
| Phosphate acetyltransferase *pta* | pta up  pta dn | GTT AAA ATC GTA TTA CCT GAA GG  GAC CCT TTT GTT GAA AAG CTT AA | 474 |
| Triosephosphate isomerase *tpi* | tpi up  tpi dn | TCG TTC ATT CTG AAC GTC GTG AA  TTT GCA CCT TCT AAC AAT TGT AC | 402 |
| Acetyl coenzyme A acetyltransferase *yqiL* | yqi up  yqi dn | CAG CAT ACA GGA CAC CTA TTG GC  CGT TGA GGA ATC GAT ACT GGA AC | 516 |

**TABLE 6**: Primers used for the detection of resistance and virulence genes

| Gene | Primer | Sequence (5’-3’) | Amplicon Size (bp) | Reference |
| --- | --- | --- | --- | --- |
| *aacA-aphD* | aacA-aphD-F  aacA-aphD-R | CCAAGAGCAATAAGGGCATACC  CACACTATCATAACCACTACCG | 222 | Vanhoof *et al*., 1994 |
| *blaZ* | blaZ-F  blaZ-R | TACA ACTGTAATATCGGAGGG  AGGAGAATAAGC AACTATATCATC | 391 | Vali *et al*., 2008 |
| *mecA* | MecA1  MecA2 | GTAGAAATGACTGAACGTCCGATA  CCAATTCCACATTGTTTCGGTCTAA | 310 | Vali *et al*., 2008 |
| *mecALGA251*  *(mecC)* | mecALGA-F  mecALGA-R | GAAAAAAAGGCTTAGAACGCCTC  GAAGATCTTTTCCGTTTTCAGC | 138 | Stegger *et al*., 2012 |
| *lukE-lukD* | LUKED-F1  LUKED-R5 | CAGATGTGAAGGGTAGTGGA  TCATTATCAGATGTTGCTGTTG | 658 | Takano *et al*., 2008 |
| *mupA* | Mup1  Mup2 | CCC ATG GCT TAC CAG TTG A  CCA TGG AGC ACT ATC CGA A | 1650 | Ramsey *et al*., 1996 & Udo *et al*., 2003 |
| *nor*A | norA+2a  norA-5 | GTAATACCAGTCTTGCCTGT  GTAATGGC TGGTCGTATCAT | 878 | Vali *et al*., 2008 |
| *pvl* | luk-PV-1  luk-PV-2 | ATCATTAGGTAAAATGTCTGGACATGATCCA  GCATCAASTGTATTGGATAGCAAAAGC | 433 | Lina G *et al*., 1999 |
| *qacA/B* | qacA/B F  qacA/B R | GCTGCATTTATGACAATGTT TG  AATCCCACCTACTAAAGCAG | 630 | Vali *et al*., 2008 |
| *qacG* | qacG-F  qacG-R | CAACAGAAATAATCGGAAC T  TACATTTAAGAGCACTACA | 275 | Vali *et al*., 2008 |
| *qacH* | qacH-F  qacH-R | ATAGTCAGTGAAGTAATAG  AGTGTGAT GATCCGAATGT | 295 | Vali *et al*., 2008 |
| *smr qacC* | smr-F  smr-R | ATAAGTACTGAAGTTATT  TTCCGAAAATGTTTAACGAAACTA | 286 | Vali *et al*., 2008 |
| *qac*C | *qacC*-F  *qacC*-R | AAACAATGCAACACCTACCACT  AACGAAACTACGCCGACTATG | 157 | Mayer *et al*., 2001 |
| *van* | vanA-1  vanA-2 | GCTGTGAGGTCGGTTGTG  GCTCGACTTCCTGATGAATACG | 111 | Okolie *et al*., 2015 |

Rapid detection, differentiation and typing of methicillin-resistant

Staphylococcus aureus harbouring either mecA or the new mecA

homologue mecA

LGA251

M. Stegger

1

, P. S. Andersen

1

, A. Kearns

2

, B. Pichon

2

, M. A. Holmes

3

, G. Edwards

4

, F. Laurent

5

, C. Teale

6

, R. Skov

1

and

A. R. Larsen

1

1) Department of Microbiological Surveillance and Research, Statens Serum Institut, Copenhagen, Denmark, 2) Microbiology Services Division, Laboratory of

Healthcare Associated Infection, Health Protection Agency, London, 3) Department of Veterinary Medicine, University of Cambridge, Cambridge, 4) Scottish

MRSA Reference Laboratory, Glasgow, UK, 5) Centre National de Reference des Staphylocoques, Lyon, France and 6) Veterinary Laboratories Agency,

Shrewsbury, UK

Rapid detection, differentiation and typing of methicillin-resistant

Staphylococcus aureus harbouring either mecA or the new mecA

homologue mecA

LGA251

M. Stegger

1

, P. S. Andersen

1

, A. Kearns

2

, B. Pichon

2

, M. A. Holmes

3

, G. Edwards

4

, F. Laurent

5

, C. Teale

6

, R. Skov

1

and

A. R. Larsen

1

1) Department of Microbiological Surveillance and Research, Statens Serum Institut, Copenhagen, Denmark, 2) Microbiology Services Division, Laboratory of

Healthcare Associated Infection, Health Protection Agency, London, 3) Department of Veterinary Medicine, University of Cambridge, Cambridge, 4) Scottish

MRSA Reference Laboratory, Glasgow, UK, 5) Centre National de Reference des Staphylocoques, Lyon, France and 6) Veterinary Laboratories Agency,

Shrewsbury, UK

Chen H.J., Hung W.C., Tseng SP, Tsai J.C., Hsueh P.R., Teng L.J. (2010). Fusidic acid resistance determinants in *Staphylococcus aureus* clinical isolates. *Antimicrob Agents Chemother*. 54 4985–4991.

Lina G., Piémont Y., Godail-Gamot F., Bes M., Peter M.O., Gauduchon V., Vandenesch F., Etienne J. (1999). Involvement of Panton-Valentine leukocidin-producing *Staphylococcus aureus* in primary skin infections and pneumonia. *Clin Infect Dis*. 29 1128–1132.

Mayer, S., Boos M., Beyer A., Fluit A. C, and Schmitz F.J. (2001). Distribution of the antiseptic resistance genes *qac*A, *qac*B, and *qac*C in 497 methicillin-resistant and –susceptible European isolates of *Staphylococcus aureus*. *J Antimicrob Chemother* 47 896–897.

Okolie CE, Wooldridge KG, Turner DP, Cockayne A, James R. (2015). Development of a heptaplex PCR assay for identification of *Staphylococcus aureus* and CoNS with simultaneous detection of virulence and antibiotic resistance genes. *BMC Microbiol*. 15 157.

Ramsey M.A., Bradley SF, Kauffman C.A., Morton T.M. (1996). Identification of chromosomal location of *mup*A gene, encoding low-level mupirocin resistance in Staphylococcal isolates. *Antimicrob Agents Chemother*. 40 2820–2823.

Stegger M., Andersen P. S., Kearns A., Pichon B., Holmes M. A., Edwards G., Laurent F., Teale C., Skov R. and Larsen A. R. (2012). Rapid detection, differentiation and typing of methicillin-resistant *Staphylococcus aureus* harbouring either *mec*A or the new *mec*A homologue mecALGA251. *Clin Microbiol Infect*.18 :395-400.

Takano T., Higuchi W., Zaraket H., Otsuka T., Baranovich T., Enany S., Saito K., Isobe H., Dohmae S., Ozaki K., Takano M., Iwao Y., Shibuya M., Okubo T., Yabe S., Shi D., Reva I., Teng L.J., Yamamoto T. (2008). Novel characteristics of community-acquired methicillin-resistant Staphylococcus aureus strains belonging to multilocus sequence type 59 in Taiwan. *Antimicrob Agents Chemother*. 52 837–845.

Udo E.E., Al-Sweih N., Noronha B.C. (2003). A chromosomal location of the mupA gene in *Staphylococcus aureus* expressing high-level mupirocin resistance. *J Antimicrob Chemother*. 51 1283–1286.

Vali L., Davies S.E., Lai L.L., Dave J., Amyes S.G. (2-008). Frequency of biocide resistance genes, antibiotic resistance and the effect of chlorhexidine exposure on clinical methicillin-resistant *Staphylococcus aureus* isolates. *J Antimicrob Chemother*. 61 524–532.

Vanhoof R, Godard C, Content J, Nyssen HJ, Hannecart-Pokorni E. (1994). Detection by polymerase chain reaction of genes encoding aminoglycoside-modifying enzymes in methicillin-resistant *Staphylococcus aureus* isolates of epidemic phage types. Belgian Study Group of Hospital Infections (GDEPIH/GOSPIZ). *J Med Microbiol*. 41 282–290
