# Supplementary material for: Characterization of Heterogeneous MRSA and MSSA with Reduced Susceptibility to Chlorhexidine in Kuwaiti Hospitals
Source: Front Microbiol. 2017 Jul 20;8:1359. doi: 10.3389/fmicb.2017.01359 (PMC5517409; doi:10.3389/fmicb.2017.01359)
Supplement: Supplementary file 2 [file DataSheet2.docx]

**Supplement 2: REAL TIME qPCR**

The qacAB positive (MRSA/MSSA) isolates identified by sequencing were selected to perform the real time qpcr. The total RNA was extracted using the RNeasy Kit and cDNA was synthesized and used as template for SYBR Green qPCR. The Ct value and melting curve analysis was used to identify the qacA and qacB genes. Further agarose gel analysis was done to confirm the amplification of a single PCR product.


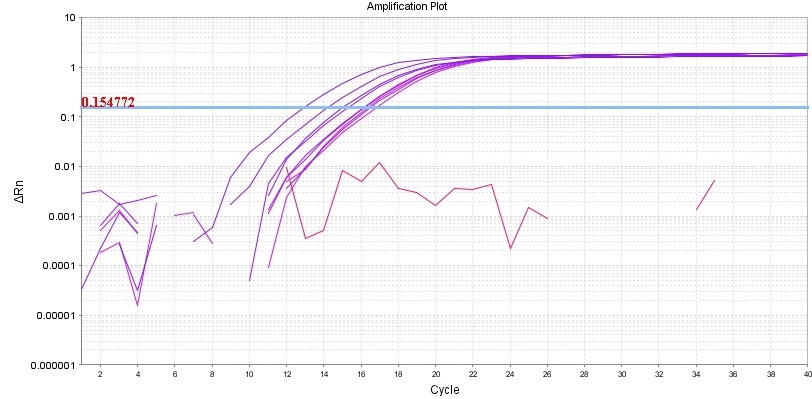

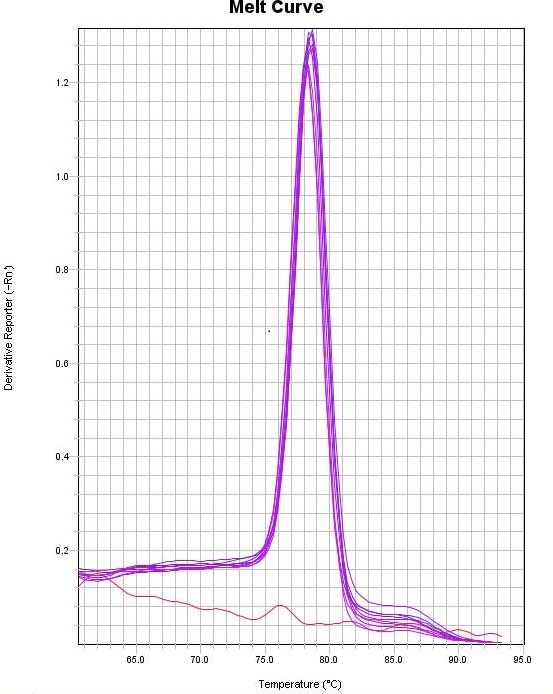


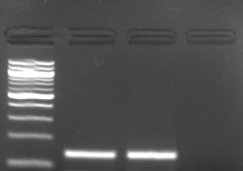
The amplification plot and the melting curve analysis showed the presence of a single pcr product of 139bp with a melting temperature of 76°C. The qacA gene was confirmed by sequencing.

L

139bp
